# Supplementary material for: Identification of photoperiod-induced specific miRNAs in the adrenal glands of Sunite sheep (Ovis aries)
Source: Front Vet Sci. 2022 Jul 22;9:888207. doi: 10.3389/fvets.2022.888207 (PMC9354845; doi:10.3389/fvets.2022.888207)
Supplement: Supplementary file 1 [file Data_Sheet_1.ZIP › Supplementary file/miRDeep2 file.zip!/miRDeep2 file/Know_Structure/SP42A2/oar-mir-485.pdf]

miRBase precursor : oar-mir-485
Total read count : 2537
oar-miR-485-5p read count : 926
oar-miR-485-3p read count : 1610
remaining reads : 1

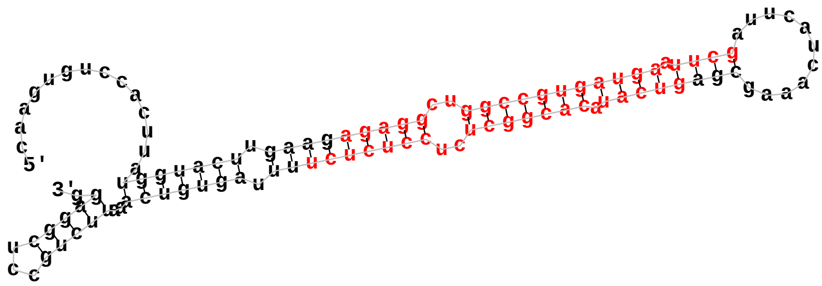

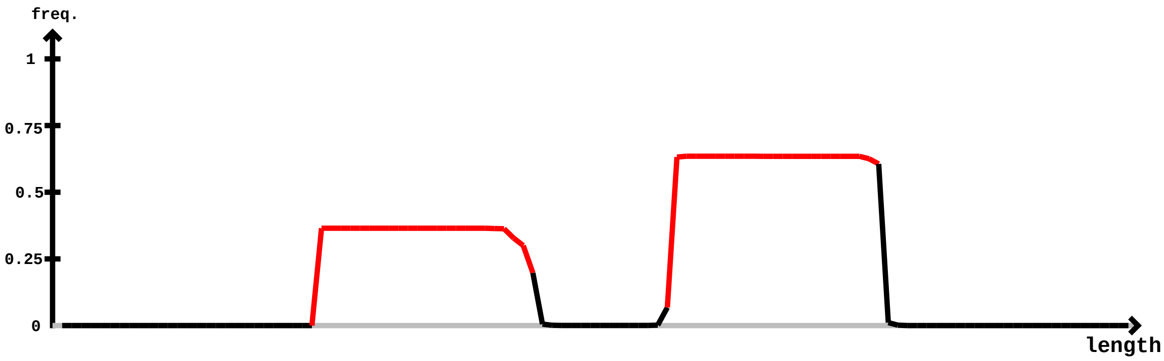

oar-miR-485-5p

oar-miR-485-3p

```
5'- caaguguccacuuaugguacuugaagagaggcuggccgugaugaauucgauucaucaaagcgagucauacacggcucuccucucuuuuagugucaaauucugccucggagg   -3'    exp
    ((((((.(((....)))))))))((((((((..(((((((((((.((((...........))))))))).)))))))..))))))))...........(((((...))))).    reads      mm         sample
    ..........................agaggcuggccgugauga...................................................................    3          0          seq
    ..........................agaggcuggccgugaugaa..................................................................    2          0          seq
    ..........................agaggcuggccgugaugaau.................................................................    89         0          seq
    ..........................agaggcuggccgugaugaauu................................................................    71         0          seq
    ..........................agaggcuggccgugaugaauuc...............................................................    260        0          seq
    ..........................agaggcuggccgugaugaauucg..............................................................    488        0          seq
    ..........................agaggcuggccgugaugaauucga.............................................................    11         0          seq
    ..........................agaggcuggccgugaugaauucgau............................................................    2          0          seq
    .................................................auucaucaaagcgagucauacac.......................................    1          0          seq
    .............................................................gagucauacacggcucuccucucu..........................    3          0          seq
    ..............................................................agucauacacggcucuccucu............................    1          0          seq
    ..............................................................agucauacacggcucuccucuc...........................    2          0          seq
    ..............................................................agucauacacggcucuccucucu..........................    162        0          seq
    ..............................................................agucauacacggcucuccucucuu.........................    2          0          seq
    ..............................................................agucauacacggcucuccucucuuu........................    1          0          seq
    ...............................................................gucauacacggcucuccucu............................    22         0          seq
    ...............................................................gucauacacggcucuccucuc...........................    46         0          seq
    ...............................................................gucauacacggcucuccucucu..........................    1338       0          seq
    ...............................................................gucauacacggcucuccucucuu.........................    22         0          seq
    ...............................................................gucauacacggcucuccucucuuu........................    3          0          seq
    ................................................................ucauacacggcucuccucuc...........................    1          0          seq
    ................................................................ucauacacggcucuccucucu..........................    7          0          seq
```
